# Rethinking Healthy Societies: A Critical Commentary on Policy Levers and Enablers: Comment on "How to Build Healthy Societies: A Thematic Analysis of Relevant Conceptual Frameworks"

**DOI:** 10.34172/ijhpm.9059

**Published:** 2025-05-27

**Authors:** Hanan Khalil

**Affiliations:** Department of Public Health, School of Psychology and Public Health, La Trobe University, Melbourne, VIC, Australia.

**Keywords:** Healthy Societies, Policy Levers, Social Determinants of Health, Political Economy, Governance, Equity

## Abstract

The pursuit of healthy societies has long been a global aspiration, yet the pathways to achieving them remain fraught with challenges. The paper "How to Build Healthy Societies: A Thematic Analysis of Relevant Conceptual Frameworks" provides an insightful thematic analysis, identifying key policy levers and enablers necessary for transformative change. This commentary critically examines the paper’s approach, highlighting the need for a more profound engagement with political and economic structures. Additionally, the commentary highlights the role of civic engagement and evidence-based policy-making in overcoming systemic inertia. Ultimately, achieving healthy societies requires a paradigm shift—one that moves beyond technical solutions towards an equity-driven and justice-oriented framework.

## Introduction

 The concept of a healthy society originated from global public health movements and policies that emphasized the broader determinants of health beyond medical care. The Alma-Ata Declaration of 1978,^1^ developed by the World Health Organization (WHO) and the United Nations International Children’s Emergency Fund, laid the foundation by advocating for Health for All through primary healthcare and intersectoral action.^2^ This idea was further strengthened by the Ottawa Charter for Health Promotion (1986), which introduced principles like equity, social justice, and creating supportive environments to improve population health.^3^ Over time, the Commission on Social Determinants of Health (2008) highlighted how social, economic, and political structures shape health outcomes, reinforcing the notion that a healthy society is built through governance, policy, and collective action rather than just healthcare interventions.^4^

 By the 2010s, the term “healthy societies” gained prominence in public health research, policy discussions, and global development frameworks.^5^ The Lancet Commission on Planetary Health (2015) linked environmental sustainability to long-term societal health, while the Sustainable Development Goals (2015) underscored the importance of health equity, education, economic stability, and climate action in fostering well-being.^6^ Today, the term is widely used by international organizations such as WHO, United Nations Development Programme, and the World Bank to describe a holistic approach that integrates governance, social policies, and economic structures to create equitable and sustainable communities. This evolution highlights how the concept of a healthy society has grown beyond its initial public health roots to encompass a multi-sectoral and global perspective on well-being.

 The paper “How to Build Healthy Societies: A Thematic Analysis of Relevant Conceptual Frameworks” offers a valuable synthesis of existing literature, proposing policy levers and enablers to guide public health interventions.^7^ The authors construct a compelling argument for intersectoral action, fiscal policies, and regulatory measures. This commentary critically evaluates their findings, interrogating the gaps in political economy, power structures, and the practical application of these frameworks. By exploring alternative models of governance, civic engagement, and systemic accountability, this discussion aims to refine and expand the discourse on what truly constitutes a “healthy society.”

## Critical Review of Policy Levers

###  Regulatory and Fiscal Measures: A Limited Lens?

 The authors highlight taxation, regulation of harmful industries, and equitable financing as primary levers for creating healthy societies. While these measures are undoubtedly essential, they assume a level of political will that is often absent in neoliberal economies. The influence of powerful corporate actors—particularly in the food, pharmaceutical, and fossil fuel industries—has historically undermined regulatory efforts, often through lobbying, legal challenges, or co-opting public health narratives. The paper does not sufficiently engage with these dynamics, leaving critical questions about enforcement and industry accountability unanswered.

 Moreover, fiscal policies such as taxes on tobacco and alcohol are presented as effective solutions, yet their regressive nature can disproportionately burden lower-income populations.^8^ A more nuanced discussion on wealth redistribution, universal basic services, and corporate taxation would provide a broader perspective on fiscal justice as a determinant of health. Furthermore, fiscal policies should consider subsidies and incentives for healthy lifestyles, including financial support for sustainable agriculture, green energy, and active transportation infrastructure, to create long-term health benefits and environmental sustainability.

###  Intersectoral Action: Breaking Silos or Reinforcing Power Asymmetries?

 The emphasis on intersectoral collaboration is a well-established principle in public health, yet its real-world implementation remains inconsistent. While the paper acknowledges the importance of Health-in-All-Policies, it does not fully explore why such initiatives often fail to gain traction. Bureaucratic resistance, sectoral fragmentation, and competing economic priorities can impede cross-sector cooperation.^9^

 Intersectoral collaboration is widely recognized as essential for addressing the social determinants of health; however, coordinating efforts across multiple sectors remains a major challenge. The paper also highlights that health is influenced by factors beyond the healthcare system, including education, housing, urban planning, and economic policies. Despite this, health ministries often lack the authority or influence to coordinate meaningful action across different governmental departments. Bureaucratic silos, competing priorities, and lack of financial and human resources further hinder effective intersectoral action. Additionally, while frameworks such as Health-in-All-Policies have been proposed, their implementation has been inconsistent, particularly in low- and middle-income countries where economic constraints and governance issues complicate cross-sector collaboration. Without clear leadership, legal mandates, and sustained political commitment, intersectoral approaches to improving health remain fragmented and largely aspirational.

 The assumption that ministries of health can lead such efforts is problematic, given that many determinants of health lie outside their jurisdiction. Alternative governance models—such as multi-stakeholder commissions with independent oversight—may offer a more viable approach to intersectoral action.^10^

 Additionally, intersectoral action must be backed by legally binding commitments, rather than remaining voluntary or advisory in nature for it to function. National and local governments should integrate health impact assessments into urban planning, environmental policies, and economic development strategies. Similarly, the inclusion of non-governmental organizations, grassroots movements, and marginalized communities in decision-making processes can enhance the effectiveness and legitimacy of intersectoral action. Table summarises categories, key issues from the paper and recommendations on how to improve them.

**Table:** A Summary of Categories, Key Issues and Recommendations

**Category**	**Key Issues**	**Recommendations**
Regulatory & fiscal measures	Corporate influence, regressive taxation, lack of accountability	Progressive taxation, wealth redistribution, corporate regulation
Intersectoral action	Bureaucratic resistance, sectoral fragmentation, governance models	Independent oversight, multi-stakeholder commissions
Redefining progress	Over-reliance on GDP, need for alternative well-being indicators	Adopt HDI, GNH, and sustainability-focused indicators
Political will	Grassroots mobilization, role of international institutions	Strengthen advocacy networks, evidence-based persuasion
Civic engagement	Role of social movements, participatory governance models	Enhance citizen oversight, participatory budgeting
Knowledge production	Decolonizing knowledge, inclusion of Global South perspectives	Support community-led research, equitable funding structures

Abbreviations: GDP, gross domestic product; HDI, Human Development Index; GNH, gross national happiness.

###  Redefining Progress: Moving Beyond Gross Domestic Product 

 A major strength of the paper is its call for a recalibration of societal progress metrics. The reliance on gross domestic product (GDP) as the primary measure of success has long been critiqued, yet alternatives such as the Human Development Index (HDI) or gross national happiness (GNH) remain underutilized.^7^

 The paper argues that alternative metrics, such as the HDI, GNH, and well-being indices, offer a more holistic view of societal health. However, transitioning to these frameworks requires a fundamental shift in policy-making, one that prioritizes equity, environmental sustainability, and long-term social welfare over short-term economic gains. Political resistance to such changes is substantial, as many governments and institutions remain invested in GDP-driven policies that favour economic expansion, often at the cost of social and environmental well-being. Without a paradigm shift in how societies measure progress, policies will continue to prioritize economic growth over the structural changes necessary to improve public health and social equity.^11^

 For a truly transformative approach, governments must adopt well-being-based economic models that prioritize quality of life, social cohesion, and ecological balance. Investment in public services such as education, universal healthcare, and affordable housing should be central to this new paradigm. Furthermore, businesses should be encouraged to adopt corporate social responsibility initiatives that align with these broader societal goals.

## The Role of Enablers: Political Will, Civic Engagement, and Knowledge Production

###  Political Will: A Missing Piece?

 Political will is identified as a crucial enabler, yet the paper does not adequately address the mechanisms through which it can be cultivated. Historical examples—such as tobacco control policies or universal healthcare reforms—suggest that political commitment often emerges from grassroots mobilization, advocacy, and evidence-based persuasion rather than top-down mandates.^12^

 Political leaders frequently prioritize short-term economic and electoral gains over long-term health investments, making it difficult to implement comprehensive health-promoting policies. Additionally, corporate influence, misinformation, and weak governance structures create barriers to holding governments and industries accountable for health outcomes. While some countries have established independent monitoring bodies and public accountability mechanisms, many still lack effective oversight, particularly in ensuring equitable policy implementation. The challenge lies in mobilizing public demand for health-oriented governance, strengthening civic engagement, and ensuring that health remains a political priority beyond individual administrations. Without systematic accountability mechanisms and empowered civil society movements, policies aimed at fostering healthy societies risk being underfunded, deprioritized, or reversed by subsequent governments.^7,13^

 While upstream policy change is critical, the commentary also recognizes the imperative for downstream, community-based interventions that can be implemented in parallel. It is acknowledged that waiting for national or global policy shifts may delay progress indefinitely. As such, the authors believe that locally feasible, context-sensitive strategies—such as municipal health charters, local taxation for health, or regional participatory governance—should be pursued concurrently. This observation is not a critique of Nambiar et al, but rather a reflection of my own perspective on how multi-level policy action can be pragmatically sequenced and aligned.

 Governments should prioritize policies that ensure political accountability, including transparency in decision-making, anti-corruption measures, and stronger regulatory frameworks for public health interventions. Political will can also be strengthened through voter education campaigns and democratic engagement initiatives that empower citizens to advocate for their right to a healthy society. The role of international institutions in shaping national policies also warrants exploration, particularly in low- and middle-income countries where external funding influences domestic priorities.

###  Civic Engagement: Mobilizing Communities for Health Equity

 The commentary strongly supports the authors’ recognition of civic engagement as an enabler. However, the paper largely frames community participation as a supplement to government action rather than a driver of systemic change. Social movements, advocacy coalitions, and rights-based approaches have historically played a pivotal role in advancing health equity. From HIV/AIDS activism to environmental justice campaigns, bottom-up pressure has often been the catalyst for policy shifts.^14^ A deeper engagement with social movement theory and participatory governance models would enhance the discussion on civic engagement as a transformative force.

 Civic engagement should also include digital activism and media campaigns to amplify marginalized voices and combat misinformation. Empowering communities through participatory budgeting and community-led decision-making processes can enhance resilience and promote a shared vision of health equity.^15^

###  Knowledge Production: Bridging Research and Policy

 The paper rightfully highlights the need for robust research to inform policy. Much of the existing literature on healthy societies is produced in high-income countries, with limited inclusion of indigenous knowledge systems, community-based research, and perspectives from the Global South. Decolonizing public health knowledge—through participatory research, co-creation methodologies, and equitable funding structures—is essential for addressing health inequities in a more holistic manner.^14^

## Conclusion

 In summary, “How to Build Healthy Societies” provides a valuable synthesis of conceptual frameworks, achieving healthy societies is not merely a matter of implementing the right policies but of fundamentally rethinking governance, economic priorities, and societal values. A justice-oriented, participatory, and equity-driven approach must underpin efforts to transform the social determinants of health. Future research should focus on case studies of successful interventions, power dynamics in policy-making, and the role of civil society in shaping health equity agendas.

## Ethical issues

 Not applicable.

## Conflicts of interest

 Author declares that she has no conflicts of interest.
